# Direct identification of a mutation in *OsSh1* causing non-shattering in a rice (*Oryza sativa* L.) mutant cultivar using whole-genome resequencing

**DOI:** 10.1038/s41598-020-71972-1

**Published:** 2020-09-10

**Authors:** Feng Li, Akira Komatsu, Miki Ohtake, Heesoo Eun, Akemi Shimizu, Hiroshi Kato

**Affiliations:** 1 Institute of Crop Science, National Agricultural and Food Research Organization (NARO), 2-1-2 Kannondai, Tsukuba, Ibaraki 305-8602 Japan; 2 Institute of Agrobiological Sciences, NARO, 1-2 Owashi, Tsukuba, Ibaraki 305-8634 Japan; 3 Institute for Agro-Environmental Sciences, NARO, 3-1-3 Kannondai, Tsukuba, Ibaraki 305-8604 Japan; 4 Radiation Breeding Division, Institute of Crop Science, NARO, Hitachi-ohmiya, Ibaraki 319-2293 Japan; 5 Genetic Resources Center, NARO, 2-1-2 Kannondai, Tsukuba, Ibaraki 305-8602 Japan

**Keywords:** Biological techniques, Genetics, Plant sciences

## Abstract

Loss of seed shattering has been regarded as a key step during crop domestication. Mutagenesis contributes to the development of novel crop cultivars with a desired seed-shattering habit in a relatively short period of time, but also to uncovering the genetic architecture of seed shattering. ‘Minamiyutaka’, a non-shattering *indica* rice cultivar, was developed from the easy-shattering cultivar ‘Moretsu’ by mutation breeding via gamma-ray irradiation. In present study, we observed significant differences in shattering habit, breaking tensile strength, and abscission zone structure between ‘Moretsu’ and ‘Minamiyutaka’. Whole-genome mutation analysis of ‘Minamiyutaka’ newly identified a 13-bp deletion causing defective splicing in exon 3 of the *OsSh1* gene which has previously been referred to as a candidate for controlling seed shattering. Using CRISPR/Cas9 gene editing, we demonstrated that loss-of-function mutation in *OsSh1* causes non-shattering in rice. Furthermore, gene expression analysis suggests that *OsSh1* may function downstream of *qSH1*, a known key gene involved in abscission zone differentiation*.* Nucleotide diversity analysis of *OsSh1* in wild rice accessions and cultivars revealed that *OsSh1* has been under strong selection during rice domestication, and a missense mutation might have contributed to the reduction of seed shattering from the wild progenitors to cultivated rice.

## Introduction

Loss or reduction of seed shattering is one of the key features in crop domestication, since it reduces yield losses from shattering and improves harvesting efficiency^1,2^. Asian cultivated rice (*Oryza sativa* L.), one of the most important cereal crops in the world, was domesticated from its Asian wild ancestor (*O. rufipogon*) more than ten thousand years ago^3,4^. In cultivated rice, the seed-shattering habit of wild rice was lost during domestication, and shattering degrees show a wide variation^5^. Generally, *indica* cultivars exhibit relatively easy shattering, whereas most *japonica* cultivars exhibit hard shattering^5^.

In rice, seeds shattering is implemented by an abscission zone in the junction of sterile lemma and pedicel^6^. The abscission zone, which is composed of one or two layers of small, isodiametrically shaped cells with thin cell walls, is formed at the young panicle development stage approximately 16–20 days before heading^7^, and gradually degrade after flowering^8^. The morphology and degradation behavior of the abscission zone differs in different rice varieties^7–9^. Seed shattering is controlled by a complex regulatory network^6,9^ and quantitative trait loci (QTLs) for seed shattering have been detected on almost all rice chromosomes^10–15^. Map-based cloning and genetic complementation experiments have revealed two domestication related mutations that facilitate the reduction of seed shattering. One mutation is a single amino acid substitution from Lysine residue to Asparagine at position 79 (K79N) in the gene *SH4*/*SHA1*, a transcription factor with an Myb3 DNA binding domain^2,16^. This mutation is responsible for the reduction of seed shattering from the wild progenitors to cultivated rice. Wild rice has a complete layer of abscission cells, while the *SH4* domestication allele contributes to absent abscission cells near the vascular bundle, leading to incomplete development of the abscission zone in cultivated rice^2^. Another mutation is a single nucleotide polymorphism (SNP) in the 5′ regulatory region of the *qSH1* gene*,* an ortholog of the *Arabidopsis* homeobox gene *REPLUMLESS* (*RPL*)^5,17^. This mutation results in the absence of abscission zone formation and thus loss of seed shattering in a subset of temperate *japonica* cultivar^5,18^. Except for *SH4* and *qSH1*, many minor QTLs involved in seed shattering have not been cloned^10–15^.

Using artificial mutagenesis, some mutants with changed seed-shattering habit were obtained and the causal genes were identified or speculated^19–24^. An insertion of a > 4-kb fragment in YABBY-like gene *OsSh1,* an ortholog of *Sh1* involved in seed shattering in sorghum, was thought to be the cause of the non-shattering phenotype in a rice mutant^19,25^. More recently, a genomic segment deletion containing *ObSH3*, an ortholog of *Sh1*, was revealed to cause the loss of seed dispersal in populations of African cultivated rice (*O. glaberrima* Steud.)^26^. Loss-of-function mutations in the *APETALA2* (*AP2*) gene, *SHAT1*^22^ or *SHH1*/*SNB*^20^, can inhibit the expression of *qSH1*, hence leading to loss of shattering. Furthermore, the natural variations in *SHH1* were speculated to be associated with the domestication and improvement of seed shattering and yield-related traits in rice^20^. In wheat, domestication-related Q gene, involved in controlling seed shattering, is also an AP2-like gene^27^. These results suggest that the regulatory network controlling seed shattering is extensively conserved in grain crops, and that mutagenesis can facilitate the uncovering of the complex genetic architecture of seed shattering.

‘Minamiyutaka’ is a non-shattering *indica* rice cultivar for whole crop silage and is broadly cultivated in Japan. ‘Minamiyutaka’ was developed from a mutant obtained through gamma-ray irradiation of an easy-shattering cultivar ‘Moretsu’^28^. The heading and maturing time, and the morphological characteristics of both cultivars are almost the same^28^. In contrast, the reported mutants have pleiotropic phenotypes such as altered development of spikelet or inflorescence, besides the reduction of seed shattering. For example, *shat1* shows spikelet and inflorescence developmental defects, *shat2* shows smaller seeds^22^, and while *ssh1* shows larger seeds^20^. We therefore hypothesized that a different causal gene was responsible for the non-shattering habit in ‘Minamiyutaka’. Identification of the causative mutation underlying the loss of shattering in ‘Minamiyutaka’ will be valuable for improving rice seed shattering without affecting other traits using molecular breeding strategies, and will also contribute to uncovering the complex regulatory pathways of seed shattering.

Recently, an approach combining bulked segregant analysis with whole-genome resequencing has dramatically accelerated the process of identifying candidate genes^20,21,29^. Alternatively, since gamma-ray-irradiation induces less than one hundred mutations in the whole genome of rice, and since, furthermore, most of them are in the intergenic and intronic regions, with only several mutations highly or moderately impacting the gene function^30^, we therefore supposed that the detection of the mutations in the whole genome and analysis of the effects of the mutations on gene function could directly identify the causal gene for an altered phenotype induced by gamma-ray-irradiation. In the present study, as expected, we successfully uncovered the putative causal gene for the loss of shattering in ‘Minamiyutaka’ with this approach and confirmed it by CRISPR (clustered regularly interspaced short palindromic repeats)/Cas9 gene editing.

## Results

### Seed-shattering characteristics in the parental cultivars

**Figure 1:**
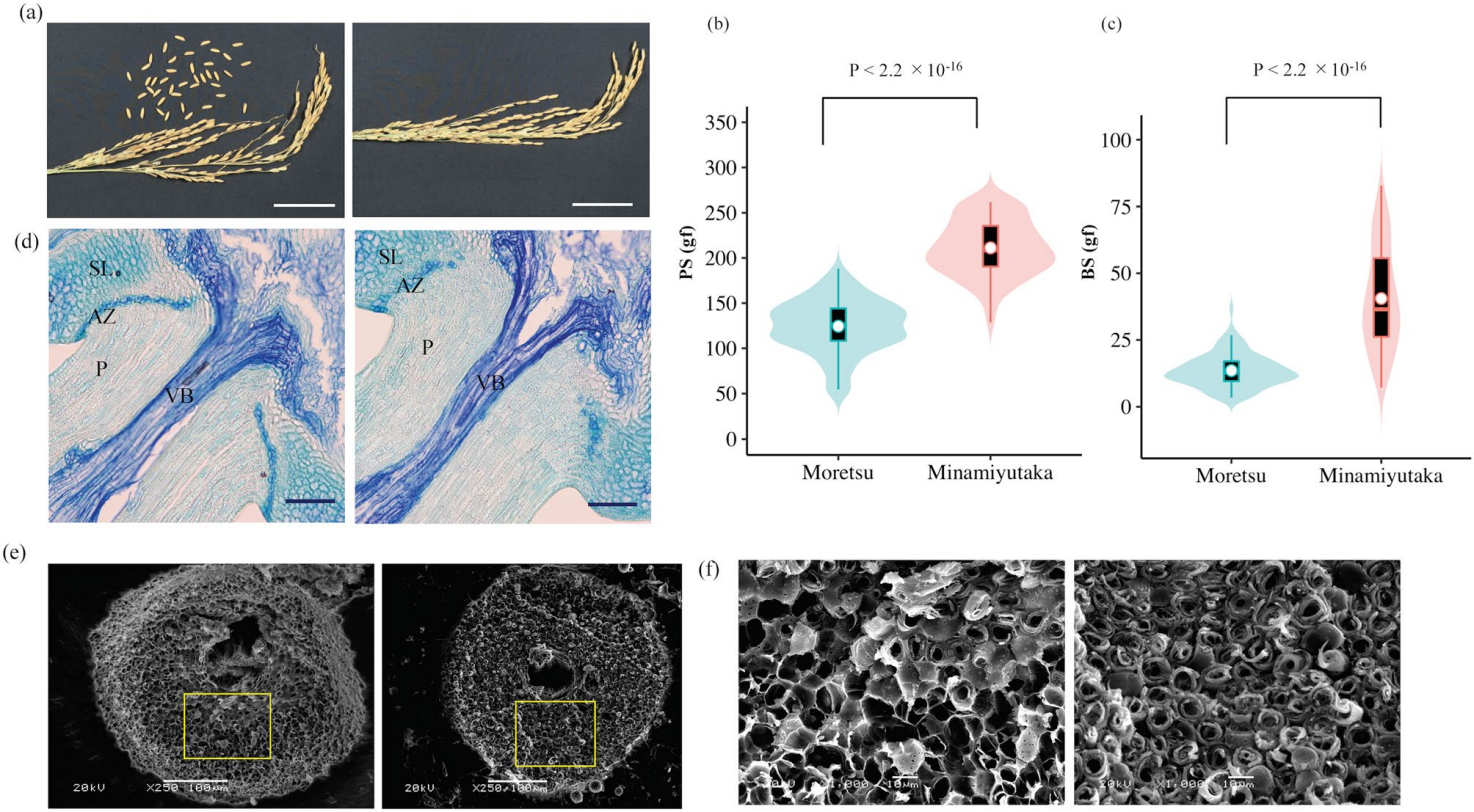
Comparison of seed shattering characteristics in the rice cultivars ‘Moretsu’ and ‘Minamiyutaka’. (**a**) Seed shattering habits of rice panicles in ‘Moretsu’ (Left) and ‘Minamiyutaka’ (Right). Photos were taken after grasping rice panicles. Bars = 5 cm. (**b**) Comparison of pulling strength (PS). *P* values were determined by Student’s *t* test. In the violin plots, the violin shape indicates the kernel-density curve, the white node in the center indicates the average value, and the black box inside the violin indicates a box-and-whisker plot. Violin plots were created using R software^62^ and ggplot2 package^63^. (**c**) Comparison of bending strength (BS). *P* values were determined by Welch’s *t* test. (**d**) Longitudinal Sects. (2-μm) across the abscission zone of ‘Moretsu’ (Left) and ‘Minamiyutaka’ (Right). The sections were stained by toluidine blue. Bars = 100 μm. *AZ* abscission zone, *P* pedicel, *SL* sterile lemma, *VB* vascular bundle. (**e**) shows scanning electron microscopy photos of the fracture surface of the grain base of ‘Moretsu’ (Left) and ‘Minamiyutaka’ (Right) after detachment of grains. Bars = 100 μm. (**f**) shows close-up scanning electron microscopy photos corresponding to the yellow boxes in (**e**). Bars = 10 μm.

The cultivar ‘Moretsu’ has an easy-shattering habit, such that up to 40 of grains were shattered after grasping a panicle by hand at the maturity stage (Fig. 1a). In contrast, almost no grains were shattered in ‘Minamiyutaka’, confirming that it is of non-shattering. Subsequently, we evaluated the pulling strength (PS) and bending strength (BS), i.e., breaking tensile strength for detachment of a seed from the pedicel by pulling and bending, respectively. PS values of ‘Moretsu’ and ‘Minamiyutaka’ were 124.4 ± 29.6 and 211.2 ± 30.9 gf, respectively, showing a significant difference (*P* < 2.2 × 10^–16^) (Fig. 1b). BS values of ‘Minamiyutaka’ were 40.5 ± 18.9 gf, approximately three times that of ‘Moretsu’ (13.5 ± 6.2 gf) (Fig. 1c). These results revealed the strong resistance to shattering in ‘Minamiyutaka’. It should be noted that the PS and BS values may also be affected by the health status of a pedicel. If a pedicel was not well developed or was damaged, its PS or BS value would be small. While we endeavored to select spikelets that appeared healthy, standard deviation was still large for all evaluated spikelets of each cultivar.

To distinguish precisely the differences in anatomical aspects of abscission zone between the two cultivars, longitudinal sections of spikelets at heading stage were observed. In ‘Moretsu’, isodiametrically shaped and thin-walled abscission zone cells were aligned completely and transversely in the basal area near sterile lemmas except in the region near the vascular bundle. In contrast, the alignment of abscission cells was discontinuous and incomplete in ‘Minamiyutaka’ (Fig. 1d).

The interface where a mature grain separates from the pedicel was observed using a scanning electron microscope (SEM). The microstructure of the surface of the grain base of ‘Moretsu’ was clearly different from that of ‘Minamiyutaka’ (Fig. 1e,f). ‘Moretsu’ was relatively smooth, but ‘Minamiyutaka’ was broken and rough. It is obvious that a stronger force was needed to remove grain from the pedicels in ‘Minamiyutaka’ than in ‘Moretsu’ and this led to a rougher surface in ‘Minamiyutaka’.

### Segregation of non-shattering phenotype

**Figure 2:**
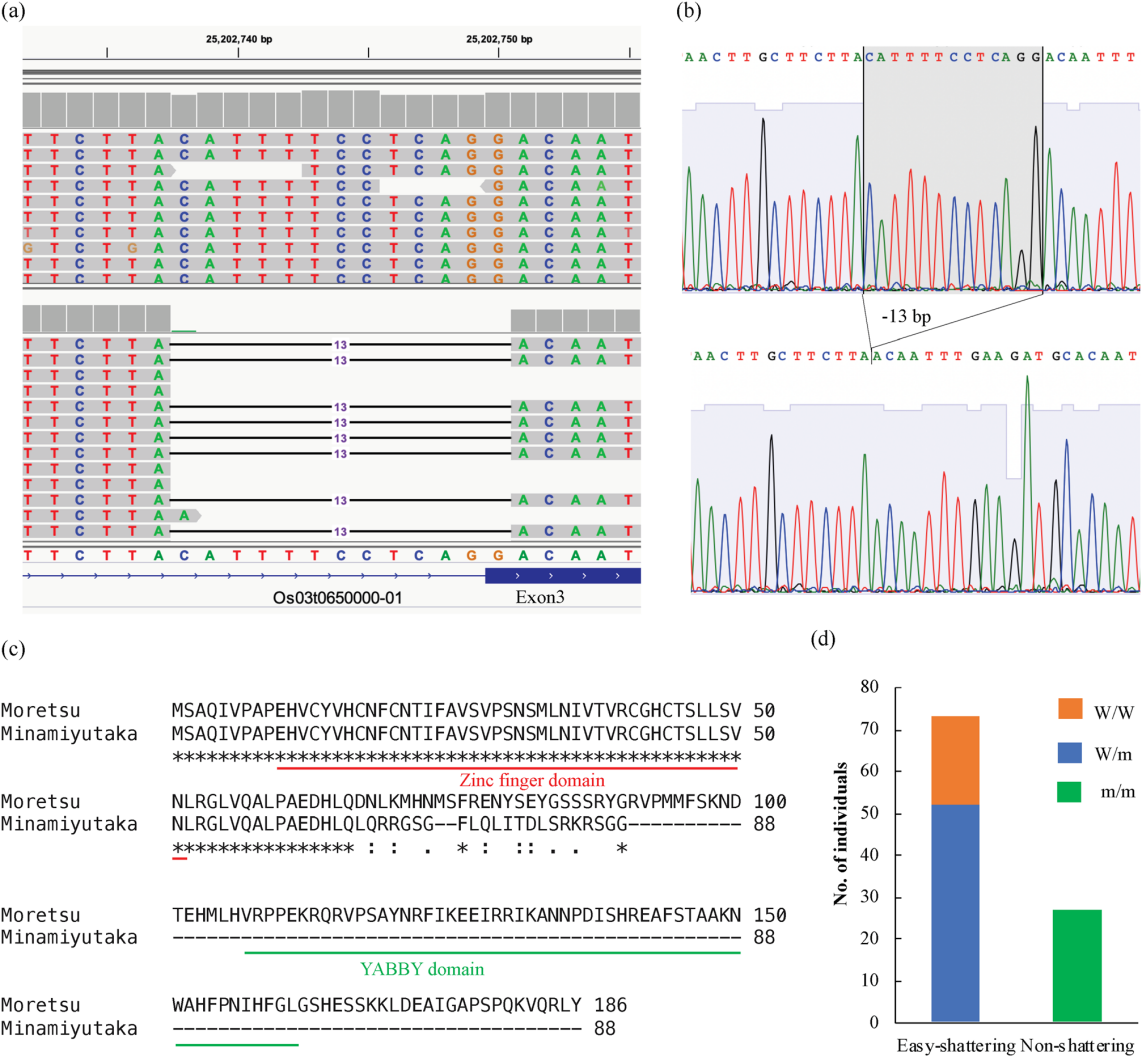
Whole-genome resequencing identified a 13-bp deletion in *OsSh1* as candidate causal mutation leading to loss of seed shattering in ‘Minamiyutaka’. (**a**) Integrative Genomics Viewer screenshot of the region containing the 13-bp deletion for ‘Moretsu’ (Upper Graph) and ‘Minamiyutaka’ (Lower Graph). Genome position and gene annotation are shown at the top and bottom, respectively. The 13-bp deletion causes defective splicing in exon 3. (**b**) Sanger sequencing chromatograms of the region containing the 13-bp deletion for ‘Moretsu’ (Upper Graph) and ‘Minamiyutaka’ (Lower Graph). (**c**) Sequence alignment of the deduced amino acid sequences of OsSh1 in ‘Moretsu’ and ‘Minamiyutaka’. The 13-bp deletion causes exon 3 skipping and a reading frameshift leading to a putative translational termination codon. Zinc finger domain and YABBY domain are underlined in red and green, respectively. (**d**) Seed shattering and genotype of the *OsSh1* gene in an F_2_ population derived from the cross between ‘Moretsu’ and ‘Minamiyutaka’. W/W (n = 21) represents a homozygous allele for ‘Moretsu’, m/m (n = 27) represents a homozygous allele for ‘Minamiyutaka’, and W/m (n = 52) for a heterozygous allele.

The shattering degrees of F_2_ plants (n = 100) derived for the cross between ‘Moretsu’ and ‘Minamiyutaka’ were evaluated by grasping panicles by hand at the maturity stage. As a result, 27 and 73 plants showed a non-shattering and an easy-shattering phenotype, respectively (Fig. 2d). This segregation ratio fits a 1:3 ratio as determined by a chi-square test (χ^2^ = 0.64, *P* = 0.42), indicating that a single recessive locus was the cause of the non-shattering phenotype in ‘Minamiyutaka’.

### Identification of a candidate causal mutation using whole-genome resequencing

Using next-generation sequencing, a total of 78.3, and 79.2 million sequence reads (each 150 bp) were obtained for ‘Moretsu’ and ‘Minamiyutaka’, respectively (Supplementary Table S1 online). After removing low-quality, unpaired and duplicated reads, about 84.0% of the clean reads were mapped to the ‘Nipponbare’ reference genome. The average coverages were 20.2 times.

Homozygous mutations including 62 SNPs, 7 deletions (< =3 bp), and 5 insertions (< =5 bp) unique to ‘Minamiyutaka’ were identified (Supplementary Table S2 online). No structural variation (SV) was detected. Mutation annotation analysis revealed only one mutation (Mutation ID: MN19, 13-bp deletion) with high impact on gene function (gene ID: Os03t0650000-01) (Fig. 2a). This mutation was validated by Sanger sequencing (Fig. 2b). In a previous study, this gene has been identified as an ortholog of *Sh1* controlling seed shattering in sorghum, and has been named *OsSh1*^25^. *OsSh1*, also called as *OsYABBY2*^31^*,* belongs to the *YABBY* gene family composed of an N-terminal zinc finger domain and a C-terminal YABBY domain (helix-loop-helix motif)^32^. Analysis of the protein coding sequence (CDS) region revealed that the 13-bp deletion caused exon 3 (127 bp) skipping and a reading frameshift leading to a putative translational termination codon (PTC) which can result in a loss of YABBY domain in ‘Minamiyutaka’ (Fig. 2c, Supplementary Fig. S1 online). We therefore inferred the 13-bp deletion in *OsSh1* to be the candidate causal mutation resulting in loss of seed shattering in ‘Minamiyutaka’.

A Cleaved Amplified Polymorphic Sequences (CAPS) marker designed based on the restriction enzyme site in the 13-bp deletion of *OsSh1* was used to perform genotyping analysis of the F_2_ plants. As a result, 21, 52, and 27 plants were homozygous wild-type, heterozygous, and homozygous mutant-type, respectively (Fig. 2d). All the wild-type and heterozygous plants exhibited the easy-shattering phenotype, whereas all the mutant-type plants were of non-shattering phenotype (Fig. 2d). These results further suggested that *OsSh1* is a strong candidate gene involved in seed shattering.

### Genetic complementation using CRISPR/Cas9-based gene editing

**Figure 3:**
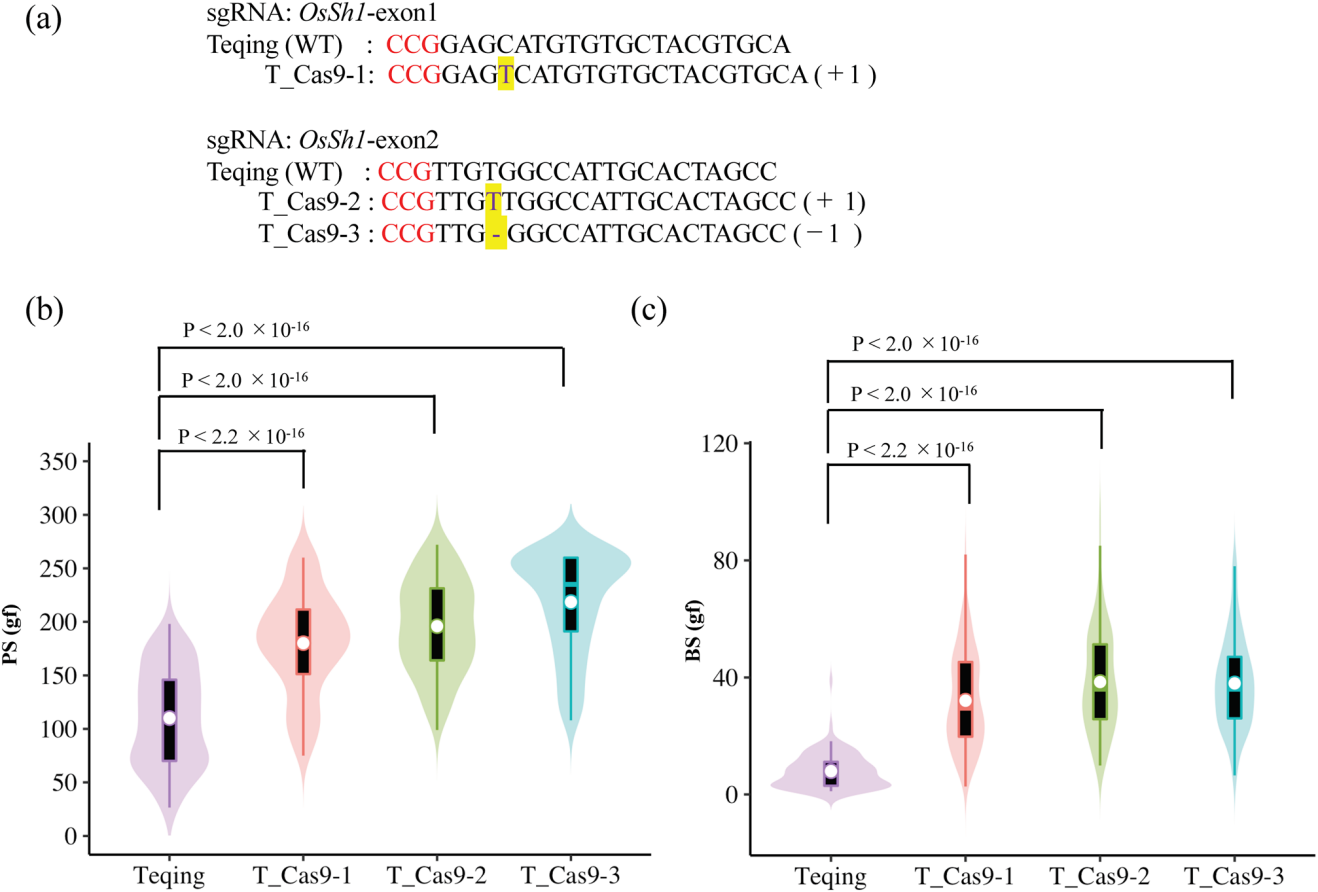
Comparison of shattering degrees among ‘Teqing’ (wild type) and *OsSh1*-edited lines in T_1_ generation at maturity stage. (**a**) Sequence alignments of the sgRNA target regions showing altered bases (in yellow) in different mutant lines. T_Cas9-1, T_Cas9-2, and T_Cas9-3 were of homozygous Cas9-free mutations derived from the T_0_ plants of 18–19-4b, 18–20-19a, and 18–26-14a (Supplementary Table S3 online), respectively. (**b**) Comparison of pulling strength (PS). P values were determined by pairwise comparisons using *t* tests with pooled SD and a Bonferroni correction. (**c**) Comparison of bending strength (BS). P values were determined by pairwise comparisons using Wilcoxon rank sum test and a Bonferroni correction. In the violin plots, the violin shape indicates the kernel-density curve, the white node in the center indicates the average value, and the black box inside the violin indicates a box-and-whisker plot. A total of 100 grains (n = 100) from four panicles were measured for each line. Violin plots were created using R software^62^ and ggplot2 package^63^.

**Figure 4:**
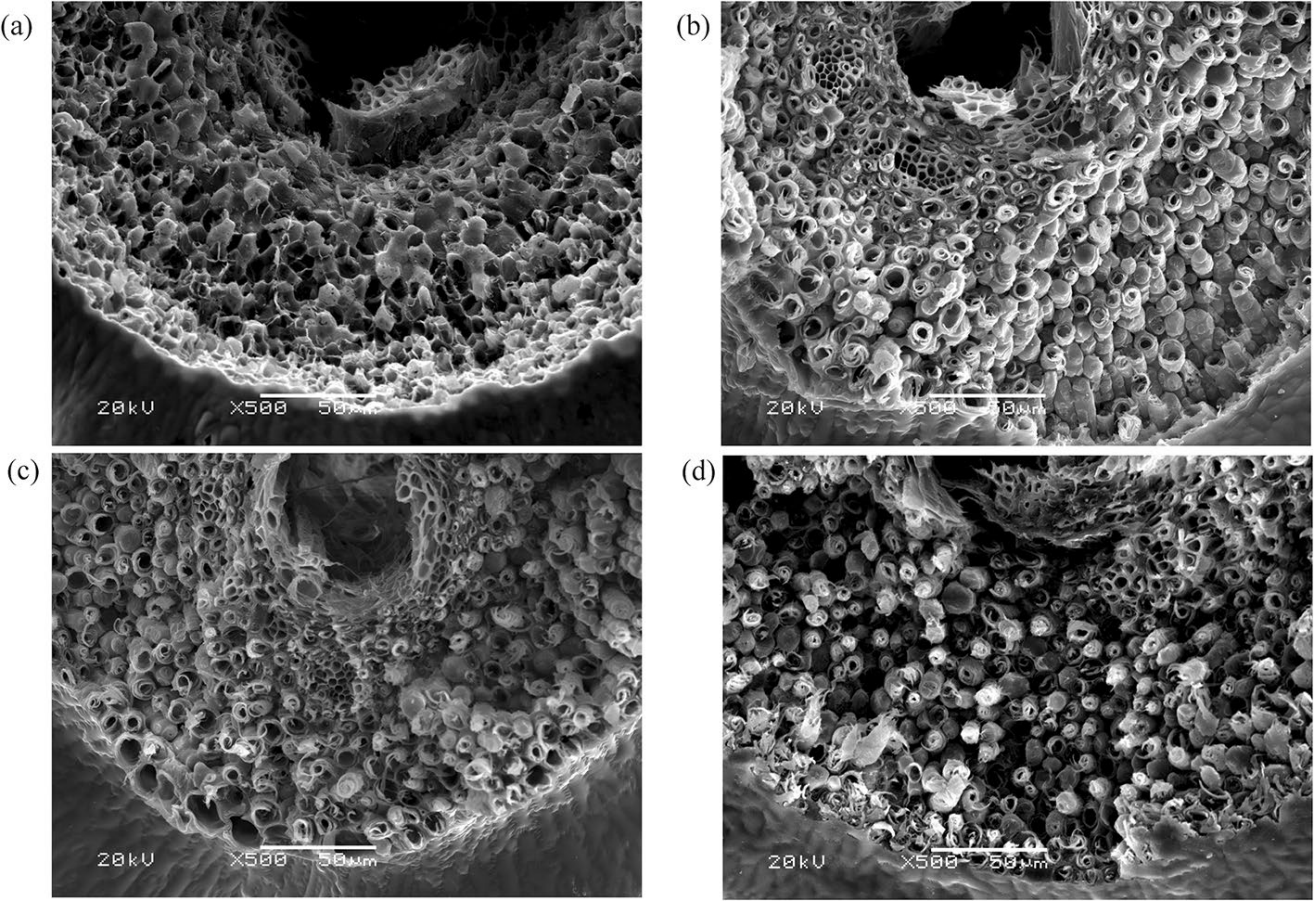
Scanning electron microscopy photos of the fracture surface of the grain base of ‘Teqing’ (**a**) and *OsSh1*-edited lines T_Cas9-1 (**b**), T_Cas9-2 (**c**) and T_Cas9-3 (**d**), bars = 50 μm.

‘Teqing’, a Chinese high-yielding *indica* rice cultivar with an easy-shattering phenotype, was used for *OsSh1* gene editing. In T_0_ plants, 8 genotypes with 11 different mutant alleles and 10 genotypes with 11 different mutant alleles were detected in the target regions of the exon 1 and exon 2 of *OsSh1*, respectively (Supplementary Table S3 online). T_0_ transgenic plants were self-pollinated and only partial T_1_ lines were grown due to space limitation in the greenhouse. For seed-shattering evaluation, we selected three independent lines, each of which had more than four plants with homozygous Cas9-free mutation (Fig. 3a). All of them have one-base InDel (deletion/insertion) in exon1 or exon2, which may cause a reading frameshift and lead to loss of gene function. PS values of the three *OsSh1*-edited lines, i.e., T_Cas9-1, T_Cas9-2 and T_Cas9-3, were 180.0 ± 47.0, 195.9 ± 43.1, and 218.5 ± 46.5gf, respectively, significantly higher than that of ‘Teqing’, 110.1 ± 45.0 gf (*P* < 2.2 × 10^–16^) (Fig. 3b). The BS values of them were 26.6 ± 11.8, 38.4 ± 16.9, and 38.0 ± 15.4 gf, respectively, approximately 2 to 5 times higher than that of ‘Teqing’ (8.0 ± 5.9 gf) (*P* < 2.2 × 10^–16^) (Fig. 3c). The fracture surface of the grain base was investigated using an SEM. We found that ‘Teqing’ had a smooth fracture surface (Fig. 4a), whereas all the *OsSh1*-edited lines had a broken and rough fracture surface (Fig. 4b–d). These results indicated that *OsSh1*-edited lines had a non-shattering habit.

### *OsSh1* expression profile

The expression profile of *OsSh1* in various rice tissues deposited in the rice gene expression database RiceXPro^33^ showed that *OsSh1* was strongly expressed in leaf blade, leaf sheath and stem rather than in root during vegetative stage (Supplementary Fig. S2 online). During reproductive stage, the *OsSh1* was mainly expressed in pistil, lemma, palea, ovary, embryo and young endosperm rather than in anther and old endosperm.

### *OsSh1 *functions downstream of *qSH1*

**Figure 5:**
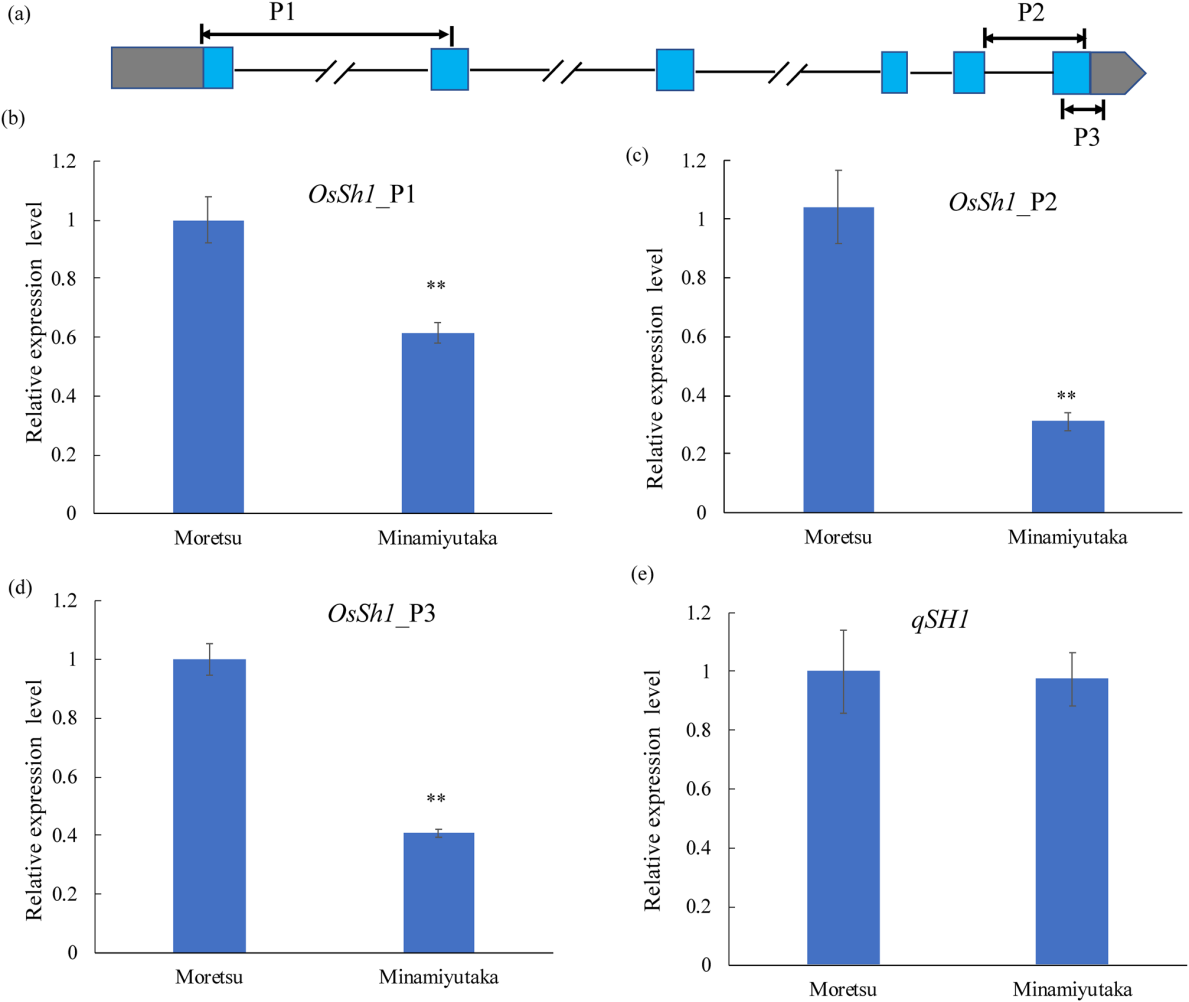
RT-qPCR analysis of *OsSh1* and *qSH1* in young panicles of ‘Moretsu’ and ‘Minamiyutaka’. (**a**) The regions of *OsSh1* were targeted using RT-qPCR with three primer sets, P1, P2, and P3, indicated by the black double-sided arrows. Note that the elements are not drawn to scale. Relative expression levels of *OsSh1* and *qSH1* are shown in (**b**)–(**e**). Data were normalized to ‘Moretsu’. Bars indicate mean values ± standard deviation (n = 4). Double asterisks denote a significant difference at *P * < 0.01 using Student's *t* test.

**Figure 6:**
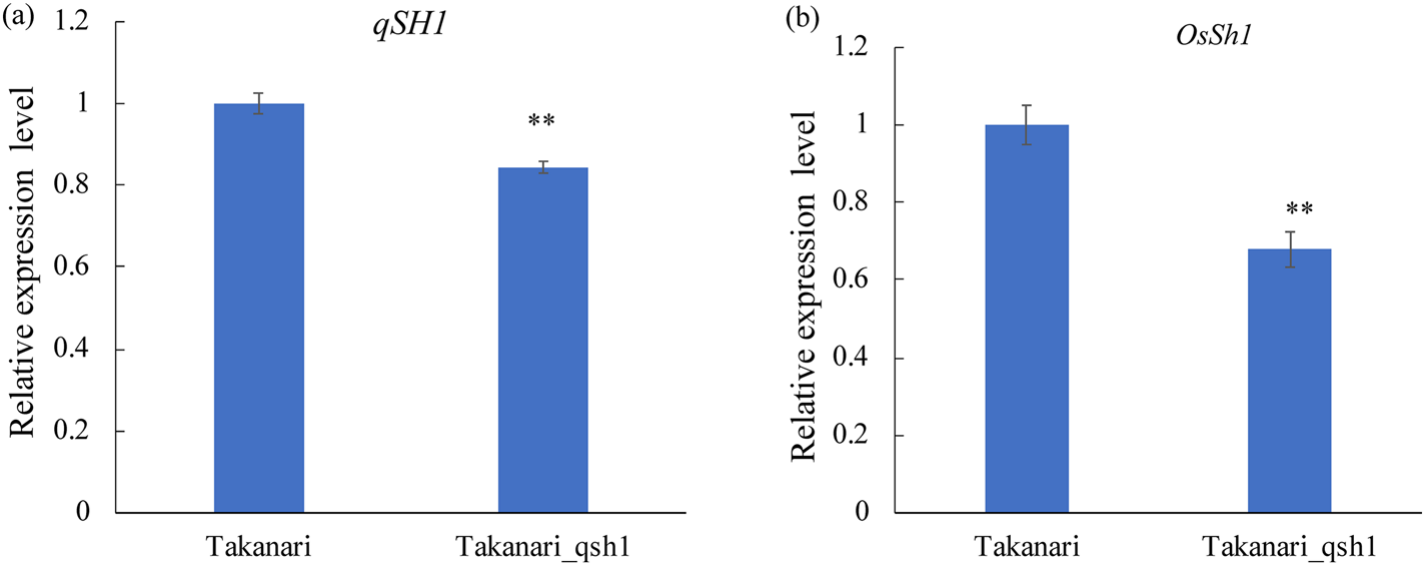
RT-qPCR analysis of *qSH1* (**a**) and *OsSh1* (**b**) in young panicles of ‘Takanari’ and ‘Takanari_qsh1’. Data were normalized to ‘Takanari’. Bars indicate mean values ± standard deviation (n = 4). Double asterisks denote a significant difference at *P * < 0.01 using Student's *t* test.

To examine the gene expression of *OsSh1* in developing panicles of ‘Moretsu’ and ‘Minamiyutaka’, we performed reverse transcription quantitative PCR (RT-qPCR) analysis using three primer sets P1, P2, and P3 (Fig. 5a). All the results showed a significantly lower level of *OsSh1* expression in ‘Minamiyutaka’ than in ‘Moretsu’ (Fig. 5b–d) (*P* < 0.01). Since *qSH1* is the key gene involved in abscission layer formation^5,22^, its transcript level was analyzed. However, no significant differences were observed between these two cultivars (Fig. 5e). These results led us to suppose that *OsSh1* might function downstream of *qSH1*, or alternatively, that *OsSh1* and *qSH1* were in different pathways, respectively. To address this, firstly, we developed a non-shattering chromosome segment substitution line (CSSL) ‘Takanari-qsh1’ which harbors a dysfunctional allele of *qSH1* derived from a *japonica* rice cultivar ‘Koshihikari’ in the ‘Takanari’ genetic background^34^. In contrast, ‘Takanari’ is an easy-shattering *indica* rice cultivar with a functional allele at *qSH1*^21,34^. PS and BS values of the ‘Takanari-qsh1’ were 188.5 ± 56.2 and 22.6 ± 13.0, respectively, significantly higher than those of ‘Takanari’, 119.1 ± 38.2 and 12.3 ± 10.5 (Supplementary Fig. S3 online), confirming the dysfunctional allele of *qSH1* causing reduction of seed shattering. Hence, we investigated the gene expressions of *OsSh1* and *qSH1* in the young panicles of these two cultivars/lines. As a result, the transcript levels of *qSH1* in ‘Takanari-qsh1’ were significantly lower than those in ‘Takanari’ (Fig. 6a), since an SNP in the 5′ regulatory region of the dysfunctional *qSH1* can decrease its expression^5^. Interestingly, *OsSh1* in ‘Takanari-qsh1’ also notably decreased as compared with ‘Takanari’ (Fig. 6b). These data suggest that *OsSh1* functions downstream of *qSH1.*

### Nucleotide diversity in *OsSh1*

**Figure 7:**
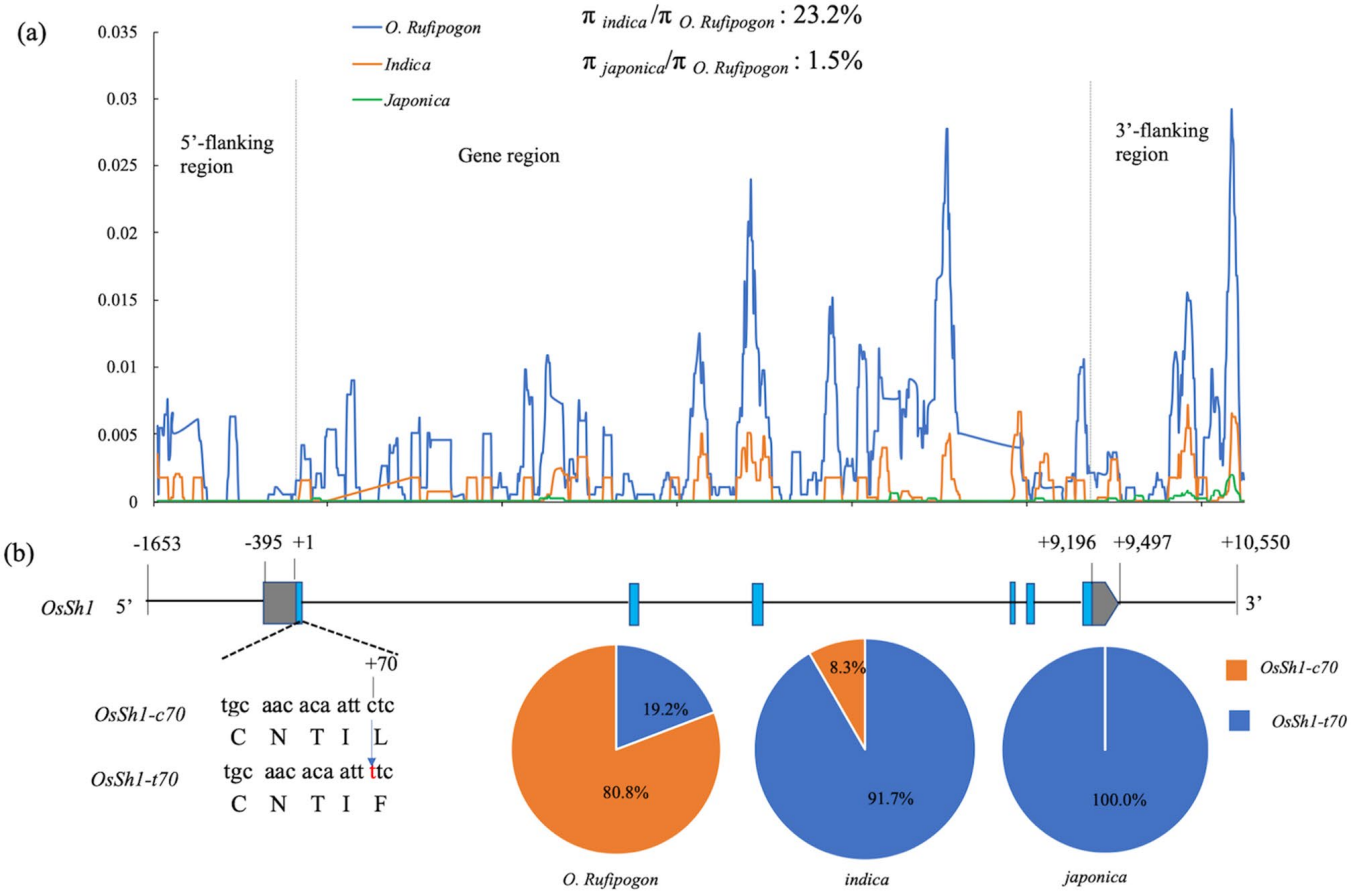
Nucleotide diversity analysis of *OsSh1* in wild and cultivated rice. The consensus sequences from 40 accessions of *O. rufipogon*, 96 *indica* varieties, 146 temperate *japonica* varieties, and 46 tropical *japonica* varieties were downloaded from TASUKE+. (**a**) Sliding-window analysis of nucleotide polymorphism (π) of *OsSh1*. The values were calculated for each sliding window of 100 bp with an increment of 10 bp. (**b**) shows a missense variant (c to t) at position + 70 nearly fixed in rice varieties. Blue boxes represent exons, grey boxes represent UTRs, and thin black lines indicate flanking regions or introns. The transcription start site of exon 1 is taken as + 1 position. Note that the elements are not drawn to scale. Pie graphs show the allele frequencies of *OsSh1-c70* and *OsSh1-t70* in *O. rufipogon*, *indica* varieties, and *japonica* varieties.

To investigate the nucleotide diversity of *OsSh1* in wild and cultivated rice, we aligned the nucleotide sequences covering the entire *OsSh1* gene (3,688 bp), a 1,653-bp 5′-flanking region, and a 1,354-bp 3′-flanking region, from 37 accessions of *O. rufipogon*, 84 *indica* varieties, 63 temperate *japonica* varieties, and 41 tropical *japonica* varieties (Supplementary Table S4 online). The nucleotide diversity (π) of *OsSh1* was the highest in wild rice, moderate in *indica* rice, and lowest in *japonica* rice (Fig. 7a). The percentages of nucleotide diversity in *indica*/*O. rufipogon* and *japonica*/*O. rufipogon* were 23.2 and 1.5%, respectively, which are far below the percentages (53.3 and 20.0%, respectively) at the whole-genome level^4,35^. Furthermore, Tajima’s D based on the *OsSh1* locus was negative and significantly (*P* < 0.01) different from neutral expectation in *O. sativa* (Supplementary Table S5 online). These results indicate that the *OsSh1* was subjected to strong directional selection during rice domestication.

Variation annotation analysis revealed that a single nucleotide variant, c to t, at position + 70 relative to the translation start site (c70t), caused a single amino acid substitution from a Leucine residue (L) to Phenylalanine (F) at position 24 (L24F) (Fig. 7b). We named these two alleles *OsSh1-c70* and *OsSh1-t70*, respectively. No other variant causing amino acid change was identified. The *OsSh1-c70* was the major allele in *O. rufipogon* (frequency = 80.8%), in contrast, *OsSh1-t70* was the major allele in *indica* rice (frequency = 91.7%), and no *OsSh1-c70* was found in *japonica* cultivars (Fig. 7b). To confirm this result, we checked the allele frequencies of *OsSh1* in 436 accessions of *O. rufipogon* (Supplementary Table S6 online), and found the allele frequencies of *OsSh1-c70* in Or-I, Or-II and Or-III to be 71.0 to 88.0%, or 81.7% overall. *OsSh1-c70* was found to be distributed extensively in the original producing area, while *OsSh1-t70* was not found in Western India, Western New Guinea, Papua New Guinea or Australia (Supplementary Fig. S4 online). Subsequently, we analyzed 1,774 *indica* cultivars and 844 *japonica* cultivars (Supplementary Table S7 online). The frequencies of *OsSh1-t70* in *indica* and *japonica* cultivars was 99.1% and 97.8%, respectively. Furthermore, *OsSh1-c70* was only found in the tropical subpopulation with a frequency at 5.0%, and was not found in subtropical and temperate subpopulations. The reason that no *OsSh1-c70* was found in the *japonica* cultivars collected in TASUKE + may be due to the limited number of tropical *japonica* varieties.

Amino acid alignment of YABBY2 proteins in different monocot and dicot plants indicated that the Zinc finger domain and YABBY domain were highly conserved (Supplementary Fig. S5 online). Leucine residue at position 24 (L24) was almost completely conserved in YABBY2 proteins of all seed plants (Supplementary Fig. S5 online), and the other YABBY genes as well^31^. These results suggested that amino acid substitution L24F in OsSh1 protein might partially affect its function in controlling seed shattering.

## Discussion

### Non-shattering phenotype in ‘Minamiyutaka’ is defect in abscission zone formation

In the present study, we found remarkable improvement of resistance to shattering in ‘Minamiyutaka’ compared to its original cultivar ‘Moretsu’ (Fig. 1). This strong shattering resistance in ‘Minamiyutaka’ may be due to the defective abscission zone formation, as in the reported non-shattering mutants such as *shat1*^22^, *shat2*^22^, and *ssh1*^20^. These results confirm that abscission zone formation is of key importance for controlling seed shattering.

### Whole-genome resequencing may directly identify the causative mutation

Genetics analysis suggested that a single recessive locus was responsible for the non-shattering phenotype in ‘Minamiyutaka’ (Fig. 2), which may facilitate cloning of the candidate gene. Through whole-genome sequencing of ‘Moretsu’ and ‘Minamiyutaka’, we newly identified a 13-bp deletion in the *OsSh1* gene of ‘Minamiyutaka’ (Fig. 2). The 13-bp deletion in *OsSh1* caused exon 3 skipping and introduced a putative PTC in the coding regions of mRNA, which can lead to generation of a non-functional OsSh1 protein (Fig. 2). The PTC-containing mRNAs are known to be degraded via nonsense-mediated mRNA decay (NMD)^36,37^. We consistently observed a notable reduction in *OsSh1* expression in ‘Minamiyutaka’ (Fig. 5), suggesting mRNAs with a PTC may be degraded. Furthermore, this 13-bp deletion was revealed to be completely associated with the non-shattering phenotype in an F_2_ population (Fig. 2). These results strongly indicate that *OsSh1* was a candidate gene. These results also suggest that whole-genome sequencing of only the mutant generated by gamma-ray irradiation and its original wild type may directly identify the candidate causal gene. This strategy may be more cost-effective and time-saving than the bulked segregant analysis^21^.

### Loss-of-function mutation in* OsSh1* can result in non-shattering phenotype

The *YABBY* gene family, specific to seed plants, is expressed in the abaxial region of leaf primordia and floral organs, and promotes abaxial cell fate and lateral organ development in *Arabidopsis*^38,39^. *YABBY* genes in poaceae such as rice, maize and wheat are not expressed in a polar manner^40–42^, suggesting that the roles of *YABBY* genes have diversified during the evolution of plants. There are 8 *YABBY* genes in rice^31^, and they also show organ-specific expression patterns^31,43^. In the present study, *OsSh1/OsYABBY2* was revealed to be expressed in all organs except for the roots and anthers, which is similar to a previous study^31^. In sorghum, *Sh1,* was identified as the gene controlling seed shattering using the mapping populations derived from the crosses between complete-shattering wild sorghum and non-shattering domesticated sorghum^25^. Interestingly, Lin et al.^25^ found that syntenic blocks containing *Sh1* correspond to the seed-shattering related QTLs in rice^44^, maize^45^, and millet^46^, indicating that *Sh1* genes for seed shattering were under parallel selection during rice, maize, and sorghum domestication. In the present study, we used the CRISPR-Cas9 genome editing system to induce loss-of-function mutations in *OsSh1* in the easy-shattering *indica* rice cultivar ‘Teqing’. The results of breaking tensile strength and microstructure revealed a remarkable improvement in resistance to shattering in the *OsSh1*-edited lines compared to the wild type (Figs. 3, 4). We therefore provide direct experimental evidence for the first time that *OsSh1* is involved in controlling seed shattering in Asian rice. Furthermore, the novel allele of *OsSh1* in ‘Minamiyutaka’ may be applied in rice breeding program.

### *OsSh1* functions downstream of *qSH1*

Thus far, the pathway controlling the development of abscission zone located between the sterile lemma and the pedicel is still unclear. It has been revealed that *qSH1* activity depends on *SHAT1*, *SH4* and *SHH1,* indicating that *qSH1* functions downstream of these genes^20,22^. In *Arabidopsis*, the *YABBY* genes such as *FILAMENTOUS FLOWER* (*FIL*) and *YABBY3* (*YAB3*) play important regulatory roles in forming stripes of valve margin tissue that allow the fruit to shatter at maturity stage^47^. Furthermore, the expression pattern of *FIL* is regulated by *RPL* (the ortholog of *qSH1* in rice)^47^. Our present study has revealed that the expression of *qSH1* is not affected by loss-of-function of *OsSh1* (Fig. 5)*,* whereas the expression of *OsSh1* decreases significantly when *qSH1* is dysfunctional (Fig. 6)*.* These results suggest that *OsSh1* may function downstream of *qSH1*, similar to the pathway in *Arabidopsis*^47^ .

### Artificial selection for *OsSh1* in rice domestication

Resequencing 50 accessions of cultivated rice and wild rice (*O. rufipogon* and *O. nivara*) suggested that *OsSh1* is one of the putative artificially selected genes^48^. We performed nucleotide diversity analysis and Tajima’s D test in many more wild rice accessions (*O. rufipogon*) and cultivars, and further confirmed very strong selection of *OsSh1* during rice domestication (Fig. 7, Supplementary Table S5 online). Furthermore, only one missense variation c70t was identified in all accessions. Since the amino acid residue L24 harbored in the *OsSh1-c70* allele was broadly conserved in seed plant species (Supplementary Fig. S5 online) as well as in the YABBY gene family^31^, *OsSh1-c70* might be an ancestral allele*.* The *OsSh1-c70* is the major allele in any ecotypes of *O. rufipogon*, and is randomly distributed geographically, whereas *OsSh1-t70* is mainly distributed in Southern China, Southeast Asia and Eastern South Asia (Supplementary Table S6 online, Supplementary Fig. S4 online), indicating that the c70t mutation might have occurred spontaneously in one of these regions and then gradually spread out from there. Interestingly, *OsSh1-c70* has become a rare allele in *indica* and tropical *japonica* cultivars which are cultivated in geographically similar regions where *O. rufipogon* is distributed, but has disappeared in subtropical and temperate *japonica* cultivars (Supplementary Table S7 online). Based on these results, we speculate possible evolutionary scenarios for *OsSh1* whereby a common ancient rice with the *OsSh1-t70* allele was first domesticated before the *indica*-*japonica* differentiation, while a few *indica* and tropical *japonica* rice accessions were crossed to local wild rice with the *OsSh1-c70* allele after differentiation. To clarify this, it might be necessary to further investigate the detailed genomic variations and DNA polymorphisms among cultivated rice containing the *OsSh1-c70* allele and wild rice.

## Materials and methods

### Materials and growth condition

‘Minamiyutaka’ and ‘Moretsu’ were crossed to generate an F_2_ population. A CSSL named ‘SL1303’, carrying a genomic region containing a dysfunctional allele of *qSH1* and a functional allele of *Semidwarf1* (*SD1*) from a *japonica* rice cultivar ‘Koshihikari’^34^ within the background of an *indica* rice cultivar ‘Takanari’, was kindly provided by Dr. Toshio Yamamoto (NARO)^34^. ‘Takanari’ is short-culm and easy-shattering, in contrast to which ‘Koshihikari’ and ‘SL1303’ show non-shattering and a long-culm phenotype. We backcrossed the ‘SL1303’ to ‘Takanari’ to develop a new non-shattering CSSL named ‘Takanari-qsh1’, whose morphological characteristics including the height of the culm is similar to that of ‘Takanari’.

### Evaluation of shattering degree

To evaluate the seed shattering of ‘Moretsu’, ‘Minamiyutaka’, gene-edited plants (including wild types), ‘Takanari’, and ‘Takanari-qsh1’, panicles from the primary tillers were harvested at maturity stage and naturally air dried for more than two weeks in a room. PS and BS were measured according to Li et al.^21^ A total of 100 to 120 grains from four panicles were measured. The seed shattering of the F_2_ plants derived from the cross between ‘Moretsu’ and ‘Minamiyutaka’ were evaluated using a method based on grasping panicles by hand at the maturity stage. In brief, three panicles from a plant were grasped by hand at one time, then the number of shattered grains then being counted. If the number of shattered grains was less than or equal to 3, this plant was regarded as non-shattering. If the number of shattered grains was more than or equal to 20, this plant was regarded as easy-shattering.

### Histological analysis

The pedicels were collected at the anthesis stage, followed by fixation in FAA (Formalin-Acetic-Alcohol) solution. After dehydration and embedding, the tissues were longitudinally sectioned into 2-μm-thick sections according to the methods described by Li et al.^21^. The sections were stained with 0.01% toluidine and were observed using an BX53 microscope (OLYMPUS, Tokyo, Japan).

### Scanning electron microscopy

The bases of mature seeds were subjected to platinum sputter coating and observed using a SEM (JEOL JSM-5610 LV, Tokyo, Japan). High resolution images were obtained in high vacuum mode at 20 kV.

### DNA extraction

The leaves were harvested from 30-day-old seedlings. For next generation sequencing, DNAs of ‘Moretsu’ and ‘Minamiyutaka’ were extracted using DNeasy Plant Maxi Kit (Qiagen Inc., Valencia, USA). For DNA marker analysis, DNAs of F_2_ plants and gene-edited plants were extracted using the simple DNA extraction method^49^.

### Whole-genome resequencing

DNA samples of ‘Moretsu’ and ‘Minamiyutaka’ were subjected to paired-end (2 × 150 bp) sequencing using an Illumina HiSeq X Ten platform. The short reads were cleaned by removing low quality reads and unpaired reads using Trimmomatic (version 0.36)^50^ with the following parameters: LEADING:10, TRAILING:10, SLIDINGWINDOW:4:20, and MINLEN:36. Next, the clean reads were aligned to the Nipponbare reference sequence (IRGSP-1.0, http://rapdb.dna.affrc.go.jp)^51^ using the mapping tool Borrows Wheeler Aligner (version 0.7.17)^52^ and indexed as BAM files using SAMtools (version 1.3.1)^53^. Duplicate fragments were then marked and eliminated with MarkDuplicates tool in Picard-Tools (Version 2.7.1.0) (https://broadinstitute.github.io/picard/). SNP, insertion, and deletion calling was performed using the HaplotypeCaller tool in GATK (Version 3.7-0)^54^. Detection of SV was performed using Pindel^55^ and Manta^56^ with default parameters.

Homozygous variations between ‘Moretsu’ and ‘Minamiyutaka’ were called with settings described below: (1) the read depth of the variant site was more than five and less than 100, (2) allele frequencies of both cultivars at a site were higher than 0.8, (3) genotypes of the two cultivars were different. To ensure the accuracy of variation detection, the candidate variations were visually confirmed using the Integrative Genomics Viewer^57^.

Variation annotation analysis was conducted based on the gene annotation of the ‘Nipponbare’ using SnpEff v4.2^58^.

### Validation of the candidate causal mutation

The causal mutation inferred by variation annotation analysis was first verified by Sanger sequencing. The methods described below have been reproduced in part from Li et al.^21^. Briefly, primers (Supplementary Table S8 online) were designed using the Primer3 program (https://bioinfo.ut.ee/primer3-0.4.0/). Purified PCR product was used for the sequencing reaction using the BigDye Terminator V3.1 cycle sequencing kit (Thermo Fisher Scientific, MA, USA), followed by sequencing on a 3730xl Genetic Analyzer (Thermo Fisher Scientific).

For analysis of co-segregation of the causal mutation with non-shattering habit in the F_2_ population, a CAPS marker (Supplementary Table S8 online) was designed based on the restriction enzyme map analysis (https://www.restrictionmapper.org). PCR amplicons were digested by the restriction enzyme BspCNI (New England Biolabs, MA, USA) at 25 °C for 60 min, and then analyzed by electrophoresis on 2.0% PrimeGel Agarose PCR-Sieve HRS (Takara) gels for 40 min at 100 V.

### Gene expression analyses

To determine the expression profile of *OsSh1* in rice plants, we analyzed the microarray data from the rice gene expression database RiceXPro (https://ricexpro.dna.affrc.go.jp)^33^. RT-qPCR analysis was conducted to compare the gene expression levels between different cultivars or lines. Total RNA was extracted using the RNeasy Plant Mini Kit (Qiagen, Hilden, Germany), from the 70–90-mm long young panicles before heading, the stage when the abscission zone is developing^59^. Each cultivar had four replications. The first-strand cDNA was synthesized from 1.0 μg of total RNA using a SuperScript III First-Strand Synthesis SuperMix for qRT-PCR (Invitrogen by Life Technologies, Carlsbad, CA, USA). *Ubiquitin* gene was used as an endogenous control to normalize detected gene expression. RT-qPCR was performed using an SYBR Green Supermix Kit (Bio-Rad, Hercules, CA, USA) on a QuantStudio 1 System (Thermo Fisher Scientific). The PCR reaction mixture (15 μl) consisted of 0.2 μM forward and reverse primers, 1 × SYBR Advantage qPCR Premix and about 10 ng cDNA. The reactions were carried out using the following qPCR protocol: 2 min pre-incubation at 95 °C followed by 40 cycles of 95 °C for 15 s and 60 °C for 1 min, and finally a dissociation run from 60 to 95 °C. Primers used for RT-qPCR experiments are listed in Supplementary Table S8 online.

### cDNA sequence analysis

A cDNA clone containing the full open reading frame (ORF) of *OsSh1* was generated by PCR using the primers listed in Supplementary Table S8 online. The PCR products were cloned into pCR-BluntII-TOPO (Invitrogen) and subjected to Sanger sequence analysis as mentioned above. The mRNA sequences of the *OsSh1* gene in ‘Moretsu’ and ‘Minamiyutaka’ have been deposited in the DNA Data Bank of Japan (DDBJ; https://www.ddbj.nig.ac.jp) with accession numbers LC522940 and LC522941, respectively. The deduced amino acid sequences were aligned using Clustal Omega with default parameters (https://www.ebi.ac.uk/Tools/msa/clustalo/). To align amino acid sequences of YABBY2 proteins in different plants, we downloaded sequences from the GenBank (https://www.ncbi.nlm.nih.gov) as follows: *O. sativa* L. ssp. *japonica* (AAX95527.1), *O. barthii* (AXM44150.1), *O. brachyantha* (XP_006650352.1), *Aegilops tauschii* ssp. *tauschii* (XP_020155668.1), *Triticum aestivum* (ABW80974.1), *Brachypodium distachyon* (XP_003561769.1), *Arabidopsis thaliana* (NP_001077490.1), *Camelina sativa* (XP_010458084.1), *Gossypium hirsutum* (XP_016683147.1), *Brassica oleracea* (XP_013638657.1). In addition, YABBY2 protein sequences in *O. sativa* L. ssp. *indica* (B8ANI4), *O. rufipogon* (A0A0E0NYX3), *O. nivara* (A0A0E0GR31), *O. punctat* (A0A0E0KGW2), *O. glumipatula* (A0A0D9ZB45) were from UniProt (https://www.uniprot.org).

### Vector construction and transformation

The sgRNA-Cas9 plant expression vector pZH_OsCas9 and the guide RNA expression vector pZK_sgRNA were kindly provided by Dr. Masaki Endo and Dr. Seiichi Toki (NARO)^60^. The oligos used in constructing the sgRNA vectors for *OsSh1* are shown in Supplementary Fig. S6a online. The easy-shattering *indica* rice cultivar ‘Teqing’ was subjected to *Agrobacterium*-mediated transformation using immature embryo-derived calli as described previously^61^. In brief, immature embryos of ‘Teqing’ from 10 to 14 days after flowering were infected by *Agrobacterium* carrying the pZH_OsU6gRNA_MMCas9 vector (Supplementary Fig. S6b online). After 5 days of co-cultivation, infected immature embryos were transferred to a fresh resting medium containing 400 mg/L carbenicillin disodium salt (Nakarai, Kyoto, Japan) to remove *Agrobacterium.* Following this, Hygromycin-resistant calli were selected over 4 weeks on a selection medium containing 400 mg/L carbenicillin disodium salt and hygromycin 30 mg/L (Wako Pure Chemicals, Osaka, Japan). Proliferating calli were then transferred to a fresh pre-regeneration medium containing 200 mg/L carbenicillin disodium salt and hygromycin 40 mg/L. After 8 days of culture, these calli were transferred to a fresh regeneration medium containing 30 mg/L hygromycin B and cultured for 2 weeks. The re-generated rice plants were grown in a closed greenhouse.

### Analysis of CRISPR-induced mutations

To analyze the mutation induced in the regenerated plants, the first or second exon of *OsSh1* were PCR amplified using the specified primers (Supplementary Table S8 online). PCR products were subjected to an *Nsp* I or *Msc*I restriction enzyme reaction, followed by agarose gel electrophoresis. PCR products showing mutation by CAPS analysis were cloned into pCR-BluntII-TOPO (Invitrogen) and subjected to Sanger sequence analysis as mentioned above.

### Statistical analysis

Statistical analyses were performed in R Software version 3.6.0^62^. Violin plots were created in the R-package ggplot2^63^. To determine the statistical significance, firstly, an F Test (two samples) or Bartlett’s test (> =3 samples) was used to test whether variances were equal for all samples. Then, if the variances were equal, Student’s *t* test (two samples) or pairwise comparisons using *t* tests with pooled SD (Standard Deviation) (> =3 samples) were performed, otherwise, Welch’s *t* test (two samples) or Wilcoxon rank sum test (> =3 samples) was performed. Finally, a Bonferroni correction was used to control for the family-wise type I error rate across the comparisons.

### DNA polymorphism analysis of candidate gene

We exported the consensus sequences covering the coding region and the flanking regions of *OsSh1* in wild and cultivated rice from TASUKE +^64^. The variant filter was set as follows: quality > =20 and depth > =4. The average number of reads that align the whole genome of an accession was set at more than eight. Nucleotide diversity analysis and test for neutral selection were performed using DnaSP (version 6.12.03)^65^. S, the number of polymorphic (segregating) sites; π, the average number of pairwise nucleotide differences per site^66^; θ, Watterson’s estimator of nucleotide polymorphism per site^67^, and Tajima’s D test^68^ were calculated. The alleles of *OsSh1* were also checked in the accessions of *O. rufipogon* in the database *OryzaGenome* (https://viewer.shigen.info/oryzagenome2detail/about/about.xhtml), and *O. sativa* in Rice SNP-Seek Database (https://snp-seek.irri.org/index.zul).

## Supplementary information

Supplementary Figures.

Supplementary Table S1.

Supplementary Table S2.

Supplementary Table S3.

Supplementary Table S4.

Supplementary Table S5.

Supplementary Table S6.

Supplementary Table S7.

Supplementary Table S8.

## Data Availability

All NGS data files will be available in the DDBJ Sequenced Read Archive under the Accession Nos. DRA009647 and DRA009648, upon acceptance of this paper.
