# Supplementary material for: Direct identification of a mutation in OsSh1 causing non-shattering in a rice (Oryza sativa L.) mutant cultivar using whole-genome resequencing
Source: Sci Rep. 2020 Sep 10;10:14936. doi: 10.1038/s41598-020-71972-1 (PMC7483440; doi:10.1038/s41598-020-71972-1)
Supplement: Supplementary file 1 — Supplementary Figures. [file 41598_2020_71972_MOESM1_ESM.docx]

**Direct identification of a mutation in *OsSh1* causing non-shattering in a rice (*Oryza sativa* L.) mutant cultivar using whole-genome resequencing**

Feng Li ^1*^, Akira Komatsu^2^, Miki Ohtake^2^, Heesoo Eun^3^, Akemi Shimizu^4^, Hiroshi Kato^5*^

^1^ Institute of Crop Science, National Agricultural and Food Research Organization (NARO), 2-1-2 Kannondai, Tsukuba, Ibaraki 305-8602, Japan. ORCID ID: 0000-0002-7706-3048

^2^ Institute of Agrobiological Sciences, NARO, 1-2 Owashi, Tsukuba, Ibaraki 305-8634, Japan

^3^ Institute for Agro-Environmental Sciences, NARO, 3-1-3 Kannondai, Tsukuba, Ibaraki 305-8604, Japan

^4^ Radiation Breeding Division, Institute of Crop Science, NARO, Hitachi-ohmiya, Ibaraki 319-2293, Japan

^5^ Genetic Resources Center, NARO, 2-1-2 Kannondai, Tsukuba, Ibaraki 305-8602, Japan

^*^ Correspondence and requests for materials should be addressed to F.L. ([rihoumail@affrc.go.jp](mailto:rihoumail@affrc.go.jp)) or H.K.(hkato@affrc.go.jp)

**Supplementary Figure**


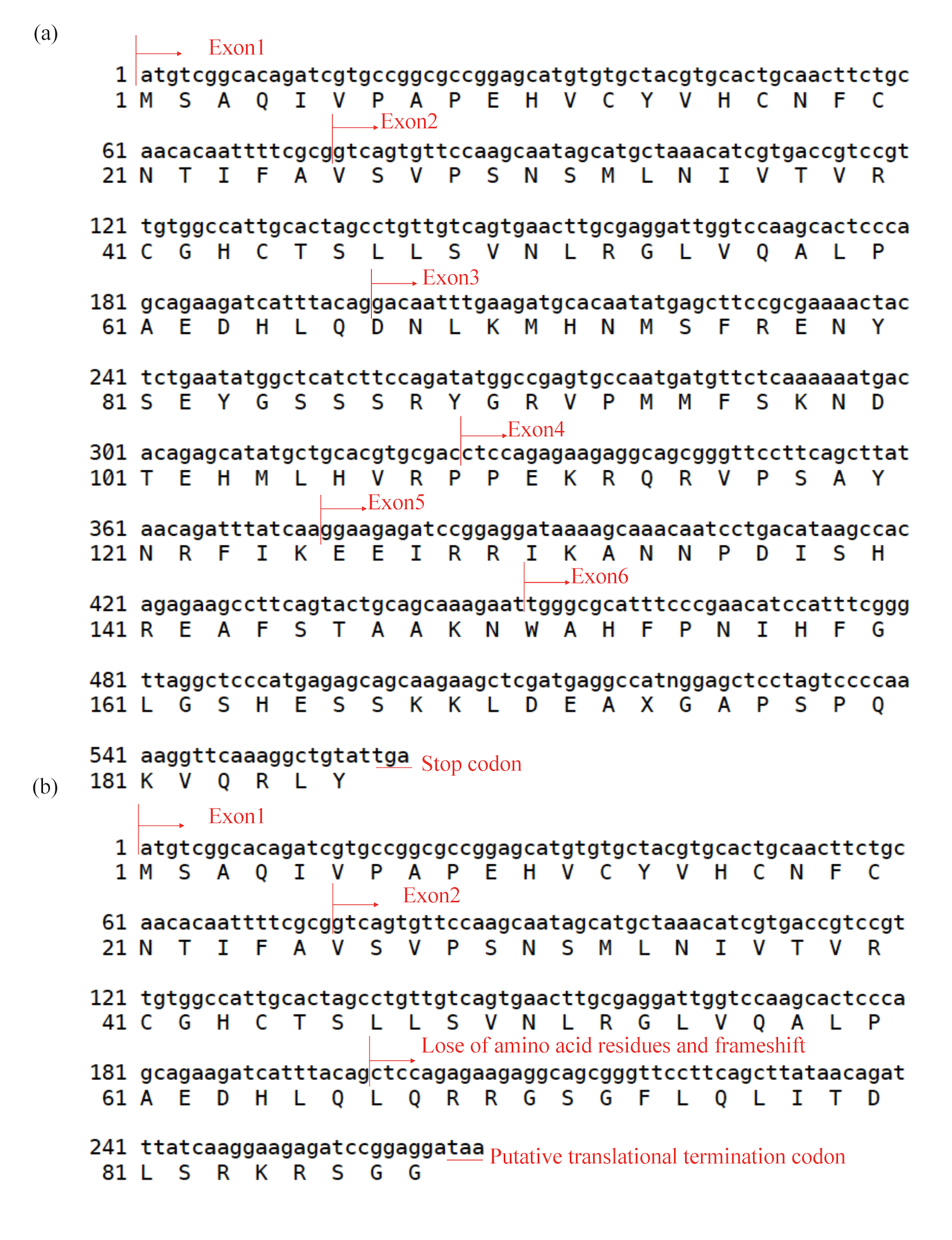


**Fig. S1** The *OsSh1* cDNA sequences and their predicted amino acid sequences in ‘Moretsu’ (a) and ‘Minamiyutaka’ (b). The boundaries for exon sequences are indicated by red vertical lines.


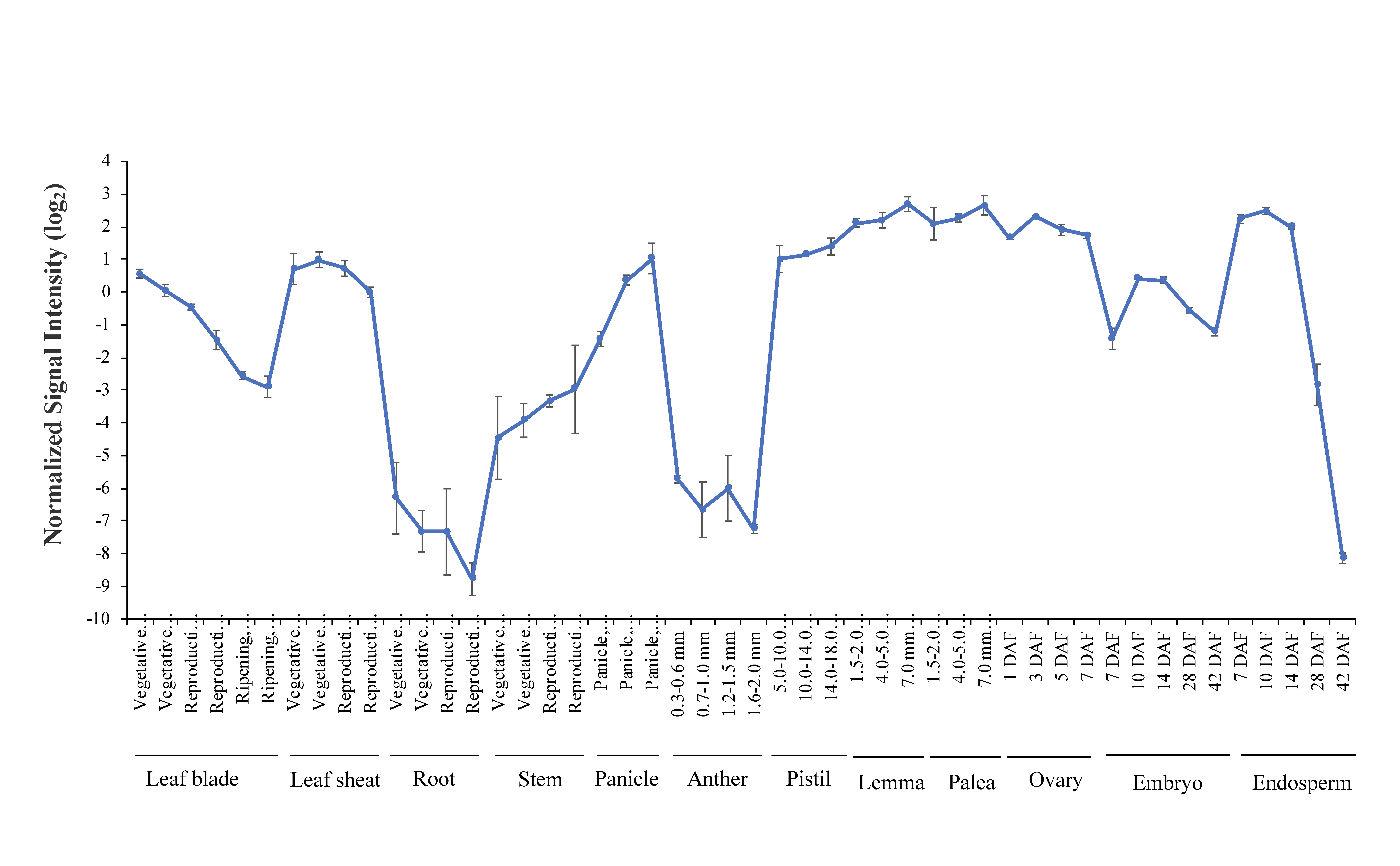


**Fig. S2** *OsSh1* expression profile based on the dataset from RiceXPro. The normalized (75 percentile) data were plotted with a standard error bar of three biological replicates.


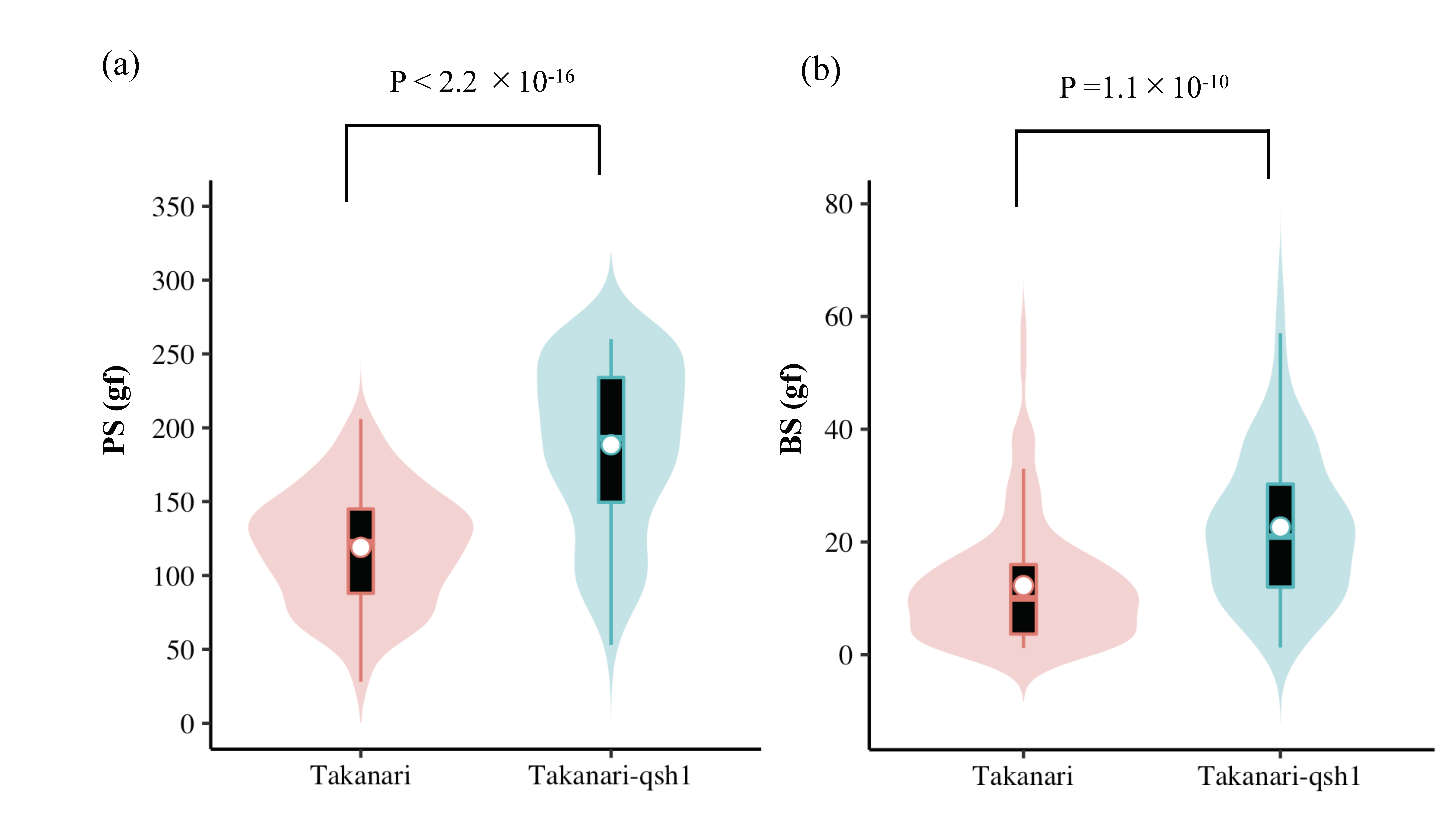


**Fig. S3** Comparison of shattering degrees in the rice cultivars ‘Takanari’ and the CSSL ‘Takanari-qsh1’. (a) Pulling strength (PS). (b) Bending strength (BS). P values were both determined by Student’s *t* test. In the violin plots, the violin shape indicates the kernel-density curve, the white node in the center indicates the average value, and the black box inside the violin indicates a box-and-whisker plot. Violin plots were created using R software (R Core Team(2019). R: A language and environment for statistical computing. R Foundation for Statistical Computing, Vienna, Austria. https://www.R-project.org/) and ggplot2 package (Wickham, H. (2016). ggplot2: elegant graphics for data analysis, Springer, New York, https://ggplot2.tidyverse.org).


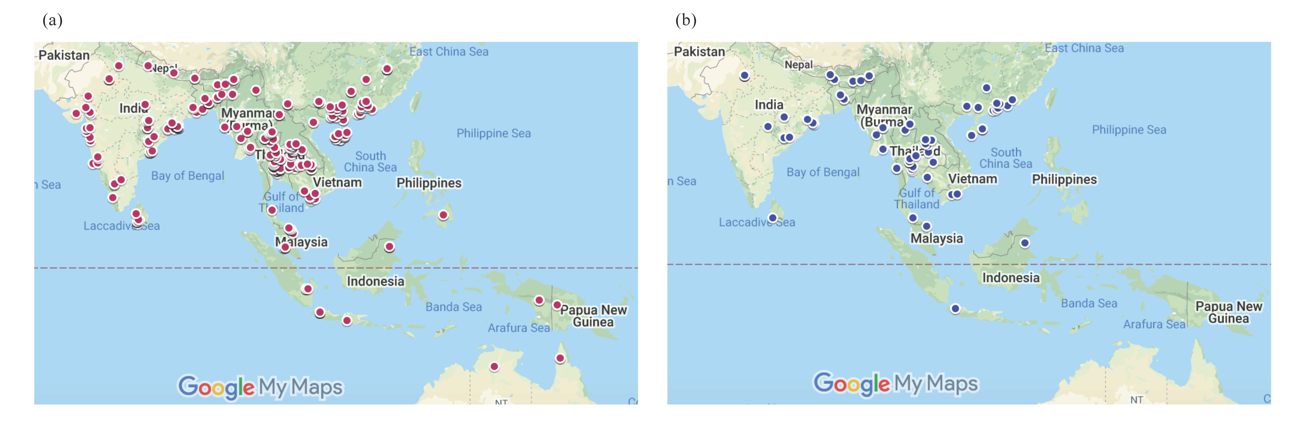


**Fig. S4** Biogeographic analysis of genetic variation at c70t site of *OsSh1* in *O. rufipogon* accessions using Google My Maps (https://www.google.com/maps/d/). (a) Red dots represent accessions carrying the *OsSh1-c70* allele. (b) Blue dots represent accessions carrying the *OsSh1-t70* allele.


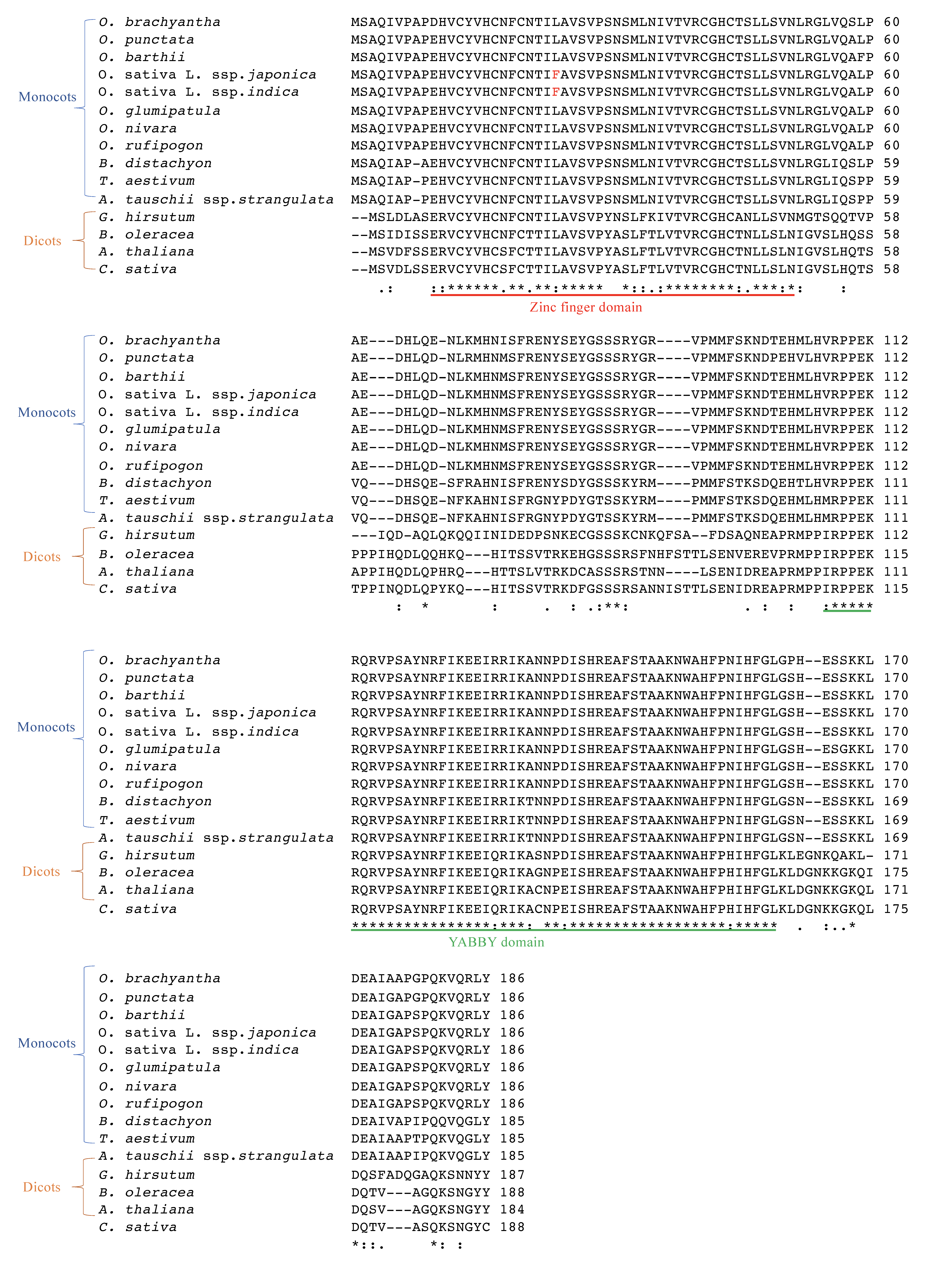
 **Fig. S5** Amino acid sequence alignment of YABBY2 proteins in different plants. Zinc finger domain and YABBY2 domain are underlined in red and green, respectively. Phenylalanine (F) at position 24 of the major allele in *japonica* and *indica* cultivars is show in red.


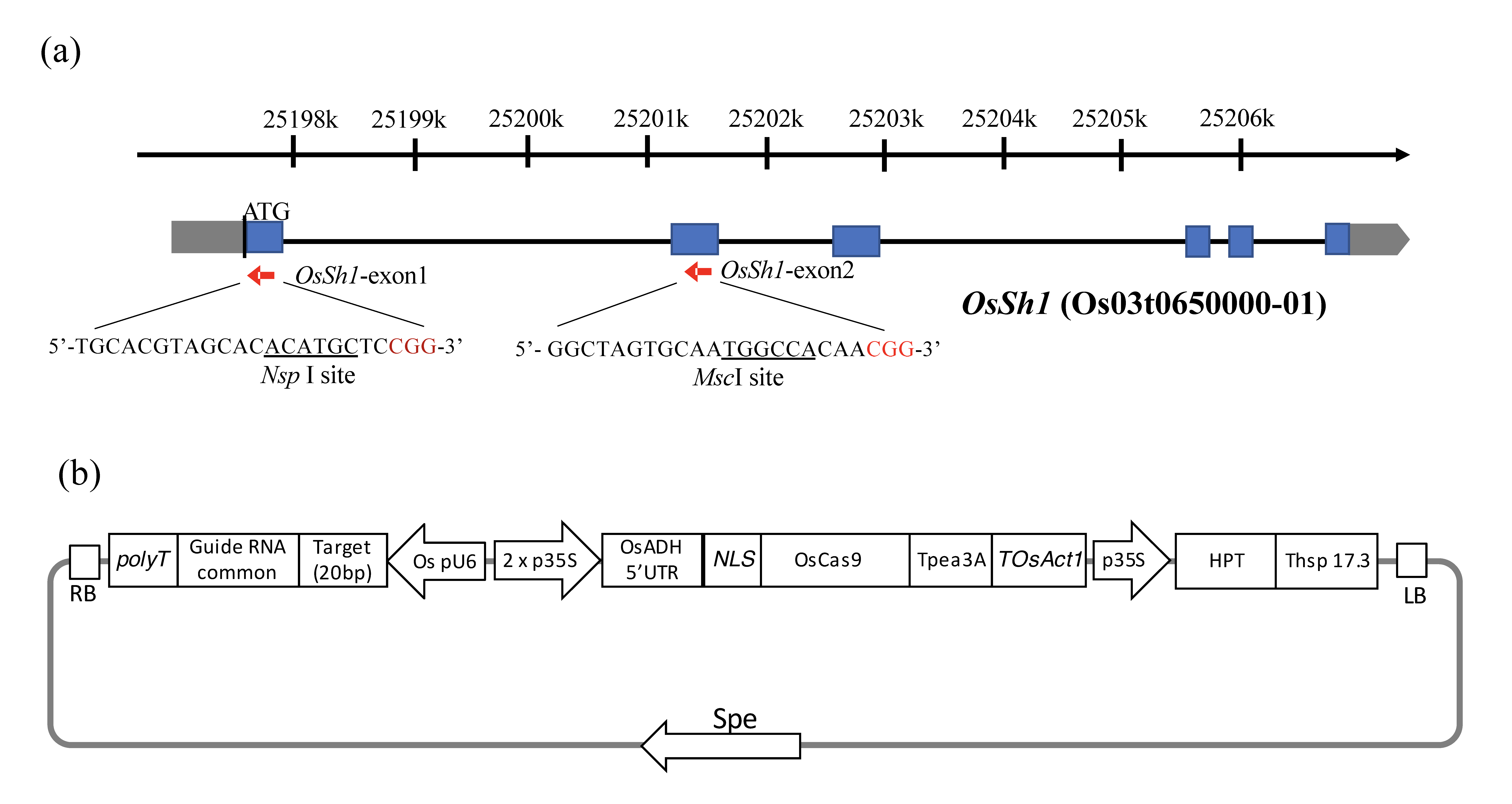


**Fig. S6** sgRNA design and vector construction for *OsSh1* editing.

(a) Schematic map of the genomic region of *OsSh1* and the two sgRNA target sites. The genome positions are based on the ‘Nipponbare’ genome sequence (IRGSP-1.0). Exon, intron, and untranslated regions are represented by the blue boxes, black lines, and gray boxes, respectively. The red arrow shows the position of the target sequence of a sgRNA. The PAM motif (NGG) is shown in red. Note that the elements are not drawn to scale. (b) Construction of pZH_OsU6gRNA_MMCas9 vector carrying sgRNA (target) under the control of the OsU6 promoter (OspU6). Expressions of *OsADH2* (alcohol dehydrogenease 2 gene), Cas9, and *HTP* (hygromycin) are driven by the Cauliflower mosaic virus 35S promoter (p35S). NLS: nuclear localization signal. Tpea3A: pea3A terminator. TOsACT1: rice actin1 terminator.
